# The EGR3 regulome of infant *KMT2A*-r acute lymphoblastic leukemia identifies differential expression of B-lineage genes predictive for outcome

**DOI:** 10.1038/s41375-023-01895-z

**Published:** 2023-04-26

**Authors:** Marius Külp, Patrizia Larghero, Julia Alten, Gunnar Cario, Cornelia Eckert, Aurélie Caye-Eude, Hélène Cavé, Tessa Schmachtel, Michela Bardini, Giovanni Cazzaniga, Paola De Lorenzo, Maria Grazia Valsecchi, Halvard Bonig, Claus Meyer, Michael A. Rieger, Rolf Marschalek

**Affiliations:** 1 Diagnostic Center of Acute Leukemia (DCAL), Institute of Pharmaceutical Biology, Goethe-University, Frankfurt am Main, Germany; 2 Department of Medicine, Hematology/Oncology, Goethe University Hospital Frankfurt, Frankfurt am Main, Germany; 3 Department of Pediatrics, University Medical Center Schleswig-Holstein, Campus Kiel, Germany; 4 Department of Pediatric Hematology and Oncology, Charité-Universitätsmedizin Berlin, Berlin, Germany; 5 Genetics Department, AP-HP, Hôpital Robert Debré, F-75019 Paris, France; 6 Université Paris Cité, Inserm U1131, Institut de recherche Saint-Louis, F-75010 Paris, France; 7 Centro Ricerca Tettamanti, Pediatrics, University of Milan-Bicocca, Fondazione Monza e Brianza per il Bambino e la sua Mamma (MBBM)/San Gerardo Hospital, Monza, Italy; 8 Genetics, School of Medicine and Surgery, University of Milan-Bicocca, Monza, Italy; 9 Statistical Section, Pediatric Clinic, University of Milan-Bicocca, Monza, Italy; 10 Center of Bioinformatics, Biostatistics and Bioimaging, University of Milan-Bicocca, Monza, Italy; 11 Institute for Transfusion Medicine and Immunohematology, Goethe University, Frankfurt am Main, Germany; 12 German Red Cross Blood Service Baden-Württemberg-Hessen, Frankfurt am Main, Germany; 13 Department of Medicine, Division of Hematology, University of Washington School of Medicine, Seattle, WA USA; 14 German Cancer Consortium (DKTK) and German Cancer Research Center (DKZF), Heidelberg, Germany; 15 Cardio-Pulmonary Institute, Frankfurt am Main, Germany

**Keywords:** Acute lymphocytic leukaemia, Acute lymphocytic leukaemia

## Abstract

*KMT2A*-rearranged acute lymphoblastic infant leukemia (*KMT2A*-r iALL) is associated with outsize risk of relapse and relapse mortality. We previously reported strong upregulation of the immediate early gene *EGR3* in *KMT2A*::*AFF1* iALL at relapse; now we provide analyses of the EGR3 regulome, which we assessed through binding and expression target analysis of an EGR3-overexpressing t(4;11) cell culture model. Our data identify EGR3 as a regulator of early B-lineage commitment. Principal component analysis of 50 *KMT2A*-r iALL patients at diagnosis and 18 at relapse provided strictly dichotomous separation of patients based on the expression of four B-lineage genes. Absence of B-lineage gene expression translates to more than two-fold poorer long-term event-free survival. In conclusion, our study presents four B-lineage genes with prognostic significance, suitable for gene expression-based risk stratification of *KMT2A*-r iALL patients.

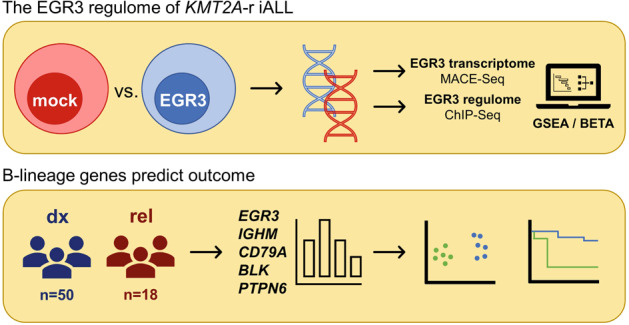

## Introduction

Seventy to eighty percent of iALL are *KMT2A*-rearranged (*KMT2A*-r); an association with inferior survival was noted and independently confirmed [1, 2]. 49% of *KMT2A*-r iALL possess the translocation t(4;11)(q21;23) generating the fusion oncogenes *KMT2A*::*AFF1* and *AFF1*::*KMT2A* [3]. On-treatment relapse is frequent and mortality is high [1, 2, 4]. Despite the successful implementation of targeted therapies in pediatric leukemia, a relapse-preventing iALL therapy remains to be established.

Our group recently reported the immediate early gene *Early Growth Response 3* (*EGR3*) as relapse-associated, with 100-fold increased *EGR3* gene expression levels at the time of relapse compared to primary diagnosis [5]. This prompted us to investigate the role of EGR3 in the context of *KMT2A*::*AFF1* iALL in more detail.

EGR3 belongs to the family of C2H2 zinc finger transcription factors with high structural and functional relation to EGR1 and EGR2 [6, 7]. EGR proteins act as direct transcriptional activators and repressors, with the ability to recruit NAB proteins as co-regulators [8–11]. Functionally, the *EGR* genes were related to neuronal development [12–16], hematopoietic stem cell quiescence [17, 18], and development of T and B cells [19–21].

Here we explore the EGR3 regulome of *KMT2A*::*AFF1* proB-ALL in detail through integration of data derived from massive analysis of cDNA ends-sequencing (MACE-Seq) and chromatin immunoprecipitation DNA-sequencing (ChIP-Seq) of an EGR3-overexpression SEM cell model. Our study identifies EGR3 as a regulator of early B-lineage specification and commitment. Additionally, gene expression and principal component analysis of 50 *KMT2A*-r iALL patients at diagnosis and 18 at relapse provides strictly bimodal clustering of patients based on the expression of the identified B-lineage genes. Absence of B-lineage gene expression translates to dismal outcome with more than two-fold poorer long-term event-free survival.

## Methods

### Cell culture

Establishment of the cell lines SEM::EGR3 and SEM::mock with Doxycycline-inducible transgene expression is described in our former study [5]. SEM cells were cultivated under sterile conditions and maintained in RPMI 1640 (RPMI-HA, Capricorn Scientific) supplemented with 10% FBS (FBS-11A, Capricorn Scientific), 2 mM L-glutamine (STA-B, Capricorn Scientific), 100 U/ml penicillin and 100 μg/ml streptomycin (PS-B, Capricorn Scientific), preheated to 37 °C prior to use. Cells were kept at 37 °C in 5% CO_2_ and a relative humidity of 95%. SEM cells were passaged twice a week keeping a density of ~1-3 × 10^6^ cells/mL. Cell lines are tested on a regular basis.

### Gene expression analysis using qRT-PCR of patient samples

Informed consent was obtained for all patients through the respective study center, which provided patient RNA. Analyzed patients displayed a proB phenotype and were diagnosed between 0 and 12 months of age (infants). RNA was extracted from peripheral blood at the day of diagnosis (dx cohort, *n* = 50) or relapse (rel cohort, *n* = 18) by the respective study center. cDNA was synthesized out of 1 µg RNA using random hexamer N6 primers and SuperScript™ II Reverse Transcriptase (18064071, invitrogen). The dx cohort is composed of *KMT2A*::*AFF1* iALL cases (Table 1), the rel cohort is composed of different *KMT2A*-r iALLs (Table 2). *IGHM, CD79A, BLK*, and *PTPN6* gene expression was measured as technical triplicates using qRT-PCR (StepOnePlus system) and ΔC_T_ mean values were calculated using *GAPDH* expression as a reference. Relative expressions were calculated as ratio (reference/target) = 2^CT(*GAPDH*) - CT(target)^ = 2^−ΔCT^. If available, clinical outcome was provided by the study centers. Used oligonucleotides are described in Table 3. The *EGR3* expression data were already assessed during our former study with the same cohorts [5]. Pearson correlations were calculated using GraphPad Prism software.

**Table 1:** Patient characteristics of the dx cohort (*n* = 50).

Patient no.	Patient age [months]	Treatment protocol	sex	Time to event (last follow-up) [months]	% *blasts*	*EGR3* ΔC_T_ Mean	*IGHM* ΔC_T_ Mean	*CD79A* ΔC_T_ Mean	*BLK* ΔC_T_ Mean	*PTPN6* ΔC_T_ Mean	*HOXA9*ΔC_T_ Mean
1	5.0	Interfant-06	F	(40.1)	49	8.1850	3.9578	2.6439	5.1517	3.8854	13.3592
2	6.0	/	F	/	82	11.2758	4.1454	2.2853	4.3700	2.1229	3.9204
3	2.0	Interfant-06	F	(23.6)	46	9.3618	4.8963	2.7340	4.4007	4.4306	12.9546
4	6.0	Interfant-06	M	(17.1)	90	11.1637	9.1284	7.8716	9.8465	10.1343	11.5783
5	10.0	/	M	/	90	8.8214	5.4178	6.2042	10.2556	9.6764	3.5974
6	2.0	Interfant-06	M	(83.6)	95	11.5618	6.2522	6.9626	8.1896	8.8812	4.9698
7	3.0	Interfant-06	F	(5.6)	96	8.5196	5.8631	2.8374	−1.4396	2.4810	11.0093
8	3.0	Interfant-06	F	(1.2)	71	8.2271	4.9521	5.1072	−0.4361	4.2495	4.9163
9	3.0	Interfant-06	M	(2.5)	85	8.0114	4.5490	3.3648	−0.8208	2.8412	4.5976
10	6.0	Interfant-06	F	9.5 (10.7)	98	12.3136	8.2192	8.1777	8.7455	8.1889	5.0818
11	0.1	/	F	/	96	11.2989	11.8824	9.8611	10.2911	9.2719	8.6964
12	1.0	/	M	/	98	11.6236	11.8944	8.6850	9.7199	9.8527	12.3443
13	12.0	/	F	/	90	9.1826	4.9885	1.9742	3.1891	−0.3517	2.2824
14	4.0	/	F	/	n/a	11.4695	6.3329	3.2521	4.4310	1.3435	11.4825
15	2.0	Interfant-06	M	6.0 (6.0)	90	13.3440	4.6279	2.6102	4.9218	1.1705	4.6973
16	5.5	Interfant-06	M	9.5 (15.0)	94	9.5330	9.9035	5.8136	6.3955	4.2272	6.2129
17	1.5	Interfant-06	F	10.0 (13.0)	92	6.2132	11.4431	8.3688	8.4548	6.1221	6.0703
18	3.0	Interfant-06	F	6.0 (11.5)	87	9.4723	9.2393	7.5200	8.7426	4.7647	2.0906
19	2.0	Interfant-06	F	10.5 (19)	96	7.2056	2.4819	2.0453	−1.9534	2.4821	7.8537
20	1.5	Interfant-06	M	26.5 (41.0)	96	11.2026	5.8739	4.8320	0.2568	6.2901	4.4627
21	6.5	Interfant-06	M	24.0 (32.8)	43	8.7817	6.5124	3.1900	0.3659	3.1614	11.2673
22	6.0	Interfant-06	F	19.5 (23.5)	94	10.0553	6.4983	2.9073	−1.3699	3.5784	11.5891
23	12.0	Interfant-06	F	32.0 (60.6)	92	8.0600	6.9642	4.0856	3.3604	1.6443	11.1226
24	9.0	Interfant-06	M	(84.9)	93	7.0569	3.3029	1.1508	2.1274	1.1842	8.7665
25	2.0	Interfant-06	F	(118.8)	93	10.2925	4.4468	1.7712	2.0883	0.4488	4.6017
26	3.0	Interfant-06	M	8.0 (108.5)	96	8.7375	7.0230	3.4687	4.0864	2.6431	13.0872
27	5.5	Interfant-06	M	(9.0)	95	9.1105	5.8320	2.9365	4.0461	1.5317	5.9626
28	5.0	Interfant-06	M	17.0 (33.0)	97	5.0456	5.0784	2.0327	4.8259	1.2226	5.9922
29	2.0	Interfant-06	F	56.0 (58.6)	90	9.3271	3.7605	3.9045	2.4719	4.2582	5.3297
30	1.5	Interfant-06	F	(146.1)	88	9.8910	3.3500	2.1610	0.6500	2.7175	5.8358
31	8.3	Interfant-06	F	(109.3)	81	6.5075	1.0567	0.0773	−0.7910	1.6433	2.2195
32	8.2	Interfant-06	M	(95.9)	92	7.2074	1.7896	2.8559	0.3826	0.8519	1.7726
33	10.7	Interfant-06	M	8.8 (8.8)	85	2.4138	-2.0719	0.3777	−0.1263	1.5011	2.6953
34	1.7	Interfant-06	F	(91.5)	93	6.2540	1.4646	1.9100	1.0432	2.1243	3.8579
35	11.7	Interfant-06	F	(73.2)	95	10.9273	4.7296	1.2813	3.3225	2.8393	2.9311
36	10.4	Interfant-06	F	(75.9)	92	10.4144	4.0039	2.3851	3.3520	2.9626	5.7541
37	2.4	Interfant-06	F	(73.8)	95	9.8438	5.2626	2.1926	4.1514	3.4033	13.3331
38	4.5	Interfant-06	M	16.1 (63.5)	87	11.4891	4.6039	1.4411	3.5708	2.5554	4.6562
39	1.1	Interfant-06	F	3.3 (15.7)	93	11.9464	6.0799	2.8532	4.2890	4.2377	12.9071
40	5.3	Interfant-06	M	(58.7)	94	10.3705	4.6305	2.2531	3.8496	3.0655	5.4284
41	6.9	Interfant-06	F	(63.2)	16	9.7053	5.4245	1.3355	3.4142	5.0462	9.2472
42	5.3	Interfant-06	M	(60.7)	88	9.5640	3.2046	−0.1283	2.8862	1.5220	3.4737
43	0.6	Interfant-06	F	5.0 (11.9)	97	8.6364	5.2214	1.5813	3.6253	2.8227	7.9122
44	1.5	Interfant-06	M	17.3 (18.3)	98	9.5745	6.0122	0.6954	3.0358	3.6119	8.1333
45	4.4	Interfant-06	M	6.6 (10.8)	82	10.2528	4.5355	0.2110	3.4874	2.4696	4.2993
46	9.1	Interfant-06	F	39.7 (45.2)	71	9.3985	5.1434	1.5751	4.8219	2.9164	3.1663
47	0.5	Interfant-06	F	8.7 (13.3)	93	9.9358	10.9029	7.9047	7.4532	8.7790	6.2701
48	6.1	AIEOP-BFM ALL 2017	F	6.6	n/a	9.4741	11.2982	8.5621	6.0722	10.3424	12.6734
49	6.1	AIEOP-BFM ALL 2017	F	8.3	n/a	10.5529	11.9934	8.9069	9.1727	8.9088	4.5847
50	10.6	/	M	/	n/a	5.1199	10.1187	9.3696	7.7286	11.0893	5.0774

Samples were taken from PB at the day of diagnosis. Gene expression values are indicated as the ΔC_T_ mean of technical triplicates.

*% blasts* Blast percentages measured at diagnosis in PB, *F* female, *M* male.

**Table 2:** Patient characteristics of the rel cohort (*n* = 18).

Patient no.	Age at primary diagnosis [months]	*KMT2A*-r partner	sex	EGR3 ΔCT Mean	IGHM ΔC_T_Mean	CD79A ΔC_T_Mean	BLK ΔC_T_Mean	PTPN6 ΔC_T_Mean
REZ1	1.3	*AFF1*	F	2.8330	3.6274	2.5294	6.2860	4.2386
REZ2	2.9	*MLLT1*	M	4.1774	8.3719	6.2586	7.3628	4.5660
REZ3	3.8	*AFF1*	F	4.3014	11.1143	7.5835	9.1950	10.0947
REZ4	2.5	*AFF1*	F	6.6574	12.0503	10.1751	11.9828	9.3876
REZ5	1.2	*AFF1*	F	1.1889	10.5701	8.1192	9.9020	9.5213
REZ6	10.7	*MLLT1*	M	5.1781	5.0754	3.5868	−0.4993	3.8987
REZ7	4.3	*MLLT1*	M	3.7298	6.0183	5.0341	−0.4709	6.2223
REZ8	3.6	*MLLT3*	F	4.7424	3.6338	4.7103	0.1660	3.3973
REZ9	9.1	*AFF1*	F	2.2762	10.9410	7.9419	8.6553	9.9990
REZ10	0.7	*AFF1*	F	1.6308	13.4663	9.1956	10.7877	10.0112
REZ11	4.4	*AFF1*	M	6.5653	10.6022	9.1016	10.0768	11.0481
REZ12	4.2	*AFF1*	M	5.0302	5.1254	3.0619	4.9896	1.7865
REZ13	2.0	*AFF1*	F	2.5849	7.4593	4.5334	4.2896	1.5519
REZ14	3.1	*AFF1*	F	8.4397	10.9710	7.2141	9.6779	1.5646
REZ15	7.6	*AFF1*	F	7.8336	4.8100	5.3276	6.5240	4.4725
REZ16	6.7	*AFF1*	M	9.5416	9.4965	5.6740	8.0000	4.4870
REZ17	5.3	*AFF1*	M	9.8667	5.2598	3.3857	−0.1229	5.3876
REZ18	7.2	*AFF1*	M	6.7247	5.9524	3.2850	0.2192	4.3320

Samples were taken from PB at relapse diagnosis. Gene expression values are indicated as the ΔC_T_ mean of technical triplicates.

*F* female, *M* male.

**Table 3:** Oligonucleotides used in this study.

Name	Sequence (5′ -> 3′)	Application
GAPDH_fwd	TTGCCCTCAACGACCACTTT	qRT-PCR
GAPDH_rev	TGGTCCAGGGGTCTTACTCC	qRT-PCR
IGHM_fwd	CATCCTGACCGTGTCCGAAG	qRT-PCR
IGHM_rev	TGGCCCACAGGTTCTCAAAG	qRT-PCR
CD79A_fwd	ACCGAATCATCACAGCCGAG	qRT-PCR
CD79A_rev	CAACCCGAGCTTCTCGTTCT	qRT-PCR
BLK_fwd	GACAGTGAATACACGGCCCA	qRT-PCR
BLK_rev	CCCGCCCATAAGTGACAACT	qRT-PCR
PTPN6_fwd	GAGGCGCAGTACAAGTTCATC	qRT-PCR
PTPN6_rev	GTTCCCGTACTCCGACTCCT	qRT-PCR

### Flow cytometry of SEM::EGR3 and SEM::mock

Cells were blocked using Human BD Fc Block^TM^ (BD) and stained with FACS antibodies (BD) according to the manufacturer’s protocol. Cells were analyzed using a BD FACSVerse^TM^. Cells of interest were gated out of all cells using FSC-H and FSC-A. Subsequently, single cells were gated and assessed for CD19, IgM and CD79A protein surface expressions with gates set using fluorescence minus one (FMO) controls. Flow cytometric analysis was performed with four biological replicates of SEM::mock and SEM::EGR3 on a BD FACSAria™ III Cell Sorter. FACS plots were created using FlowJo™ Software. Antibodies used for flow cytometry are described in Table 4.

**Table 4:** Antibodies used in this study.

Antibody	Source	Identifier
PE Mouse Anti-Human CD79a Monoclonal Antibody	BD	BD Biosciences Cat# 563777, RRID: AB_2738423
BV421 Mouse Anti-Human IgM Monoclonal Antibody	BD	BD Biosciences Cat# 562618, RRID: AB_2737681
APC-H7 Mouse Anti-Human CD19 Monoclonal Antibody	BD	BD Biosciences Cat# 560177, RRID: AB_1645470

### Massive analysis of cDNA ends-sequencing (MACE-Seq)

MACE-Seq is 3′ single end mRNA sequencing enabling high resolution transcription profiling of RNA extracted from three biological replicates of SEM::mock and SEM::EGR3 48 h after induction with Doxycycline 1 µg/mL. Screen tape analysis of RNA was performed using the bioanalyzer Agilent 2200 TapeStation assessing the RNA integrity number (RIN). RIN values above 8.5 were considered as tolerable. MACE-Seq of extracted RNAs was performed by GenXPro GmbH. MACE-Seq data are available at GEO with accession code GSE225710.

### Chromatin immunoprecipitation DNA-sequencing (ChIP-Seq)

ChIP-Seq data of SEM::EGR3 immunoprecipitated with an α-FLAG antibody in comparison to input were already obtained during our former study (GSE205652). ChIP-Seq data are available at GEO with accession code GSE205652.

### Statistics and data analysis

Appropriate statistical tests were performed within *DeSeq2*, *GSEA,* or *BETA plus* algorithms in context of differential expression and gene set enrichment analyses of MACE-Seq data or binding and expression target analysis of ChIP- and MACE-Seq data, respectively. The level of significance is indicated by p values (*DeSeq2*), false discovery rates (*GSEA*) or rank products (*BETA plus*). Used expression and binding data meet the demands of respective algorithms and associated statistical tests in terms of normality and equal-variance assumptions. The phenotypic populations assessed by flow cytometry were compared using two-tailed *t* tests performed using GraphPad Prism 9.5.0 software. Principal component analyses (PCA) were conducted as singular value decompositions using ClustVis [22] with applied unit variance scaling for rows.

Sample sizes were chosen depending on experimental context. qRT-PCR was performed as technical triplicates of 68 patient samples in total. MACE-Seq was performed using three biological triplicates of SEM::mock and SEM::EGR3 RNA. Flow cytometric analysis was performed using four biological replicates of SEM::mock and SEM::EGR3.

Gene set enrichment analysis (GSEA) was conducted using GSEA 4.2.3 according to the developer’s protocol [23].

Integration of MACE-Seq and ChIP-Seq data as well as transcription factor motif scanning was performed using the online tool BETA plus according to the developer’s protocol [24].

The 3890 up- and 3107 downregulated direct EGR3 target genes identified with BETA were uploaded to the PANTHER 17.0 classification system [25]. The functional classification considering protein class, biological function/gene ontology (GO) term, and chromosomal location was plotted using Microsoft Excel as net plots.

Survival analysis was performed using GraphPad Prism 9.4.1. Event-free survival (EFS) was defined as the time from diagnosis to first failure including induction failure, relapse, death, or second malignant neoplasm according to the Interfant-99 protocol [2]. Time was censored at last follow-up if no events were observed. Curves were computed with the Kaplan–Meier estimator, standard errors (SE) with the Greenwood formula, and curves were compared with the log-rank test.

## Results

### Overexpression of *EGR3* in the *KMT2A::AFF1* proB cell line SEM upregulates B-lineage specification and commitment genes

We recently identified *EGR3* as a relapse-associated factor in *KMT2A*-r iALL, whose gene expression is ~100-fold increased in patients at relapse (rel) compared to primary diagnosis (dx) [5]. Surprisingly, re-analysis of the Leukemia MILE study data involving 2096 patient samples using the online database BloodSpot revealed B cell malignancies to be characterized by a decreased *EGR3* gene expression in comparison to healthy bone marrow (BM) (Supplementary Fig. 1A) [26, 27]. Regarding healthy hematopoietic cells, the BloodSpot DMAP dataset demonstrates low *EGR3* expression in proB cells which strongly elevates with differentiation to naïve B cells and their progeny (Supplementary Fig. 1B) [28].

Accordingly, the inducible *EGR3* overexpression SEM cell model (SEM::EGR3) of our previous study represents the cellular identity of leukemic blasts in relapsed patients, with a proB phenotype, a strongly increased *EGR3* gene expression, and the *KMT2A*::*AFF1* genotype. To ascertain the role of EGR3 in disease progression and relapse, we performed massive analysis of cDNA ends-sequencing (MACE-Seq) of SEM::EGR3 and the corresponding empty vector control cell line SEM::mock. MACE-Seq and differential gene expression analysis using DeSeq2 identified 10,645 differentially expressed genes between SEM::EGR3 and SEM::mock, referred to as the EGR3 transcriptome. 6082 genes were up- and 4563 downregulated (log2> or <0, *p* < 0.05). A set of B-lineage genes was found to be strongly overexpressed (*IGHM* (log2fc = 11.42), *CD79A* (log2fc = 6.84), *BLK* (log2fc = 8.55), *PTPN6* (log2fc = 7.16), *CD22* (log2fc = 6.42), *CD19* (log2fc = 5.21*, IGLL1* (log2fc = 8.91).

**Fig. 1:**
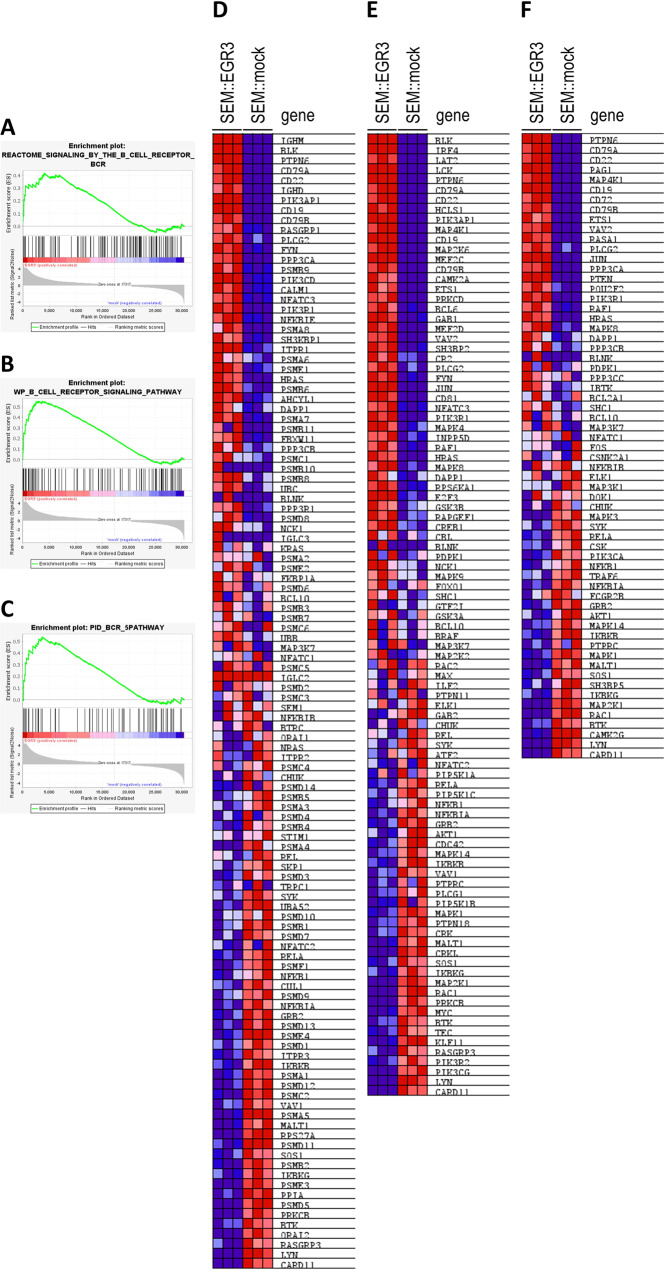
Gene set enrichment analysis (GSEA) of SEM::EGR3 and SEM::mock. Enrichment plots and corresponding heatmaps of the gene sets ‘REACTOME-SIGNALING-BY-THE-B-CELL-RECEPTOR-BCR’ (**A**, **D**), ‘WP_B_CELL-RECEPTOR-SIGNALING-PATHWAY’ (**B**, **E**) and ‘PID_BCR_5PATHWAY’ (**C**, **F**).

Gene set enrichment analysis (GSEA) was used to functionally characterize the EGR3 transcriptome considering 13,938 gene sets, of which 9910 were significantly enriched in the phenotype EGR3 at a nominal *p* value below 0.01. ‘Signaling by the B cell receptor’ (Reactome, R-HSA-983705) was identified as the highest ranked gene set (NES = 2.96, FDR = 0.050) (Table 5, Fig. 1A, D). This was corroborated by the B cell receptor (BCR) signaling gene sets of Wiki Pathways (NES = 2.73, FDR = 0.050) (Fig. 1B, E) and the Pathway Interaction Database (NES = 2.61, FDR = 0.050) (Fig. 1C, F) being at ranks four and 18, respectively [29–31]. The top scored upregulated genes in the three sets were *IGHM*, *CD79A*, *BLK*, *PTPN6*, *CD22,* and *CD19* (Fig. 1D–F). Importantly, also the pre-BCR surrogate light chain gene *IGLL1* was found to be upregulated upon EGR3 overexpression (log2fc = 8.91, *p* = 5.03 × 10^−16^). In summary, GSEA identified genes involved in BCR signaling as important targets of the EGR3 induced transcriptional profile.

**Table 5:** Top fifty gene sets enriched in phenotype EGR3.

rank	gene set	size	ES	NES	FDR *q*-val	rank at max
1	REACTOME_SIGNALING_BY_THE_B_CELL_RECEPTOR_BCR	114	0.41	2.96	0.05	4040
2	LI_WILMS_TUMOR_VS_FETAL_KIDNEY_1_DN	164	0.41	2.79	0.05	1819
3	GNF2_PTPRC	67	0.62	2.74	0.05	2657
4	WP_B_CELL_RECEPTOR_SIGNALING_PATHWAY	97	0.55	2.73	0.05	3716
5	GOBP_POSITIVE_REGULATION_OF_INTERFERON_BETA_PRODUCTION	37	0.52	2.73	0.05	2366
6	GOBP_ATP_METABOLIC_PROCESS	201	0.38	2.71	0.05	4185
7	HP_MYOCLONUS	348	0.42	2.71	0.05	4350
8	YAP1_UP	43	0.51	2.7	0.05	4532
9	GOBP_INTERFERON_BETA_PRODUCTION	55	0.51	2.67	0.05	2366
10	REACTOME_DDX58_IFIH1_MEDIATED_INDUCTION_OF_INTERFERON_ALPHA_BETA	68	0.46	2.66	0.05	2201
11	ALCALA_APOPTOSIS	85	0.41	2.66	0.05	2052
12	DER_IFN_BETA_RESPONSE_UP	103	0.43	2.66	0.05	3809
13	BROWNE_INTERFERON_RESPONSIVE_GENES	64	0.57	2.66	0.05	3071
14	GNF2_VAV1	35	0.58	2.66	0.05	3195
15	HP_BRITTLE_HAIR	40	0.52	2.65	0.05	1355
16	GOBP_DEFENSE_RESPONSE_TO_SYMBIONT	257	0.43	2.63	0.05	3422
17	NGUYEN_NOTCH1_TARGETS_DN	82	0.5	2.62	0.05	3342
18	PID_BCR_5PATHWAY	63	0.54	2.61	0.05	3716
19	HP_CHOREOATHETOSIS	99	0.41	2.61	0.05	3007
20	REACTOME_SARS_COV_INFECTIONS	355	0.34	2.61	0.05	6457
21	HP_MEMORY_IMPAIRMENT	139	0.43	2.61	0.05	3896
22	GOBP_RESPONSE_TO_VIRUS	347	0.44	2.59	0.05	3494
23	HALLMARK_INTERFERON_ALPHA_RESPONSE	96	0.46	2.56	0.05	4121
24	GOBP_REGULATION_OF_INTRACELLULAR_TRANSPORT	320	0.35	2.56	0.05	6271
25	HP_NEOPLASM_OF_THE_LIVER	124	0.49	2.55	0.05	5154
26	WP_APOPTOSIS	85	0.46	2.55	0.05	3921
27	REACTOME_TAK1_DEPENDENT_IKK_AND_NF_KAPPA_B_ACTIVATION	43	0.53	2.54	0.05	2041
28	HP_ECTOPIC_CALCIFICATION	209	0.44	2.54	0.05	3466
29	HP_ABNORMAL_CNS_MYELINATION	307	0.36	2.53	0.05	6718
30	BOQUEST_STEM_CELL_DN	206	0.51	2.52	0.05	2711
31	HP_WEAKNESS_DUE_TO_UPPER_MOTOR_NEURON_DYSFUNCTION	469	0.39	2.52	0.05	4528
32	HP_ACUTE_LEUKEMIA	105	0.41	2.52	0.05	5158
33	REACTOME_RHO_GTPASE_CYCLE	436	0.37	2.52	0.05	4897
34	HOEBEKE_LYMPHOID_STEM_CELL_UP	92	0.44	2.51	0.05	2556
35	GOBP_REGULATION_OF_VIRAL_GENOME_REPLICATION	82	0.45	2.51	0.05	3466
36	REACTOME_INTERLEUKIN_1_FAMILY_SIGNALING	144	0.39	2.51	0.05	2041
37	REACTOME_CYTOSOLIC_SENSORS_OF_PATHOGEN_ASSOCIATED_DNA	62	0.41	2.51	0.05	2201
38	WANG_TARGETS_OF_MLL_CBP_FUSION_UP	43	0.49	2.51	0.05	4346
39	GYORFFY_DOXORUBICIN_RESISTANCE	33	0.65	2.51	0.05	2036
40	HALLMARK_ALLOGRAFT_REJECTION	179	0.55	2.51	0.05	3064
41	GOBP_ACTIVATION_OF_GTPASE_ACTIVITY	109	0.49	2.51	0.05	2641
42	GNF2_MYD88	59	0.5	2.49	0.05	2926
43	MODULE_256	58	0.46	2.49	0.05	4870
44	HP_CHRONIC_DIARRHEA	75	0.48	2.48	0.05	3992
45	HP_ABNORMALITY_OF_CARDIOVASCULAR_SYSTEM_ELECTROPHYSIOLOGY	449	0.4	2.48	0.05	3898
46	YAGI_AML_WITH_T_8_21_TRANSLOCATION	351	0.39	2.48	0.05	5568
47	HP_PREMATURE_GRAYING_OF_HAIR	39	0.51	2.48	0.05	1796
48	HP_CNS_DEMYELINATION	39	0.61	2.48	0.05	4378
49	HP_SHORTENING_OF_ALL_DISTAL_PHALANGES_OF_THE_FINGERS	39	0.52	2.48	0.05	5023
50	GOBP_REGULATION_OF_GENERATION_OF_PRECURSOR_METABOLITES_AND_ENERGY	122	0.45	2.48	0.05	4933

*ES* enrichment score, *NES* nominal ES, *FDR* false discovery rate.

**Fig. 2:**
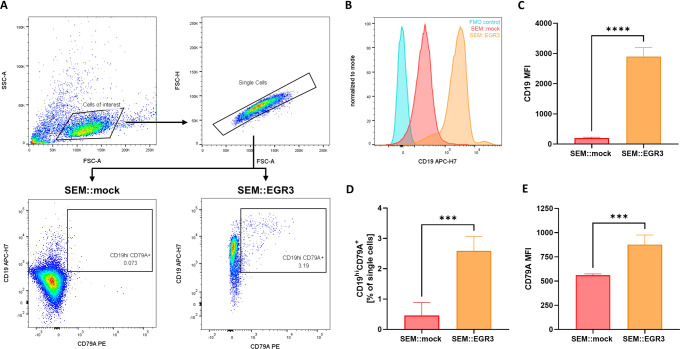
Flow cytometric analysis of SEM::EGR3 and SEM::mock. **A** Gating strategy. Single cells were gated out of cells of interest for assessment of CD19 and CD79A surface expressions. Analyses were performed based on four biological replicates. **B** Exemplary histogram of CD19 fluorescence intensities of a full-fluorescence-minus-anti CD19 (FMO) control (blue), SEM::mock (red) and SEM::EGR3 (orange). Plots display the respective single cell populations and were normalized to mode. **C** CD19 median fluorescence intensities (MFI) of SEM::mock and SEM::EGR3 singlets. Significance was tested using a two-tailed *t* test (*p* < 0.0001). Error bars indicate standard deviation. **D** Mean percentages of the CD19^hi^CD79A^+^ population relative to singlets. Error bars indicate standard deviation and significance was tested using a two-tailed *t* test (*p* = 0.0006). **E** CD79A median fluorescence intensities (MFI) of the CD19^hi^CD79A^+^ populations of SEM::mock and SEM::EGR3. Significance was tested using a two-tailed *t* test (*p* = 0.0008). Error bars indicate standard deviation.

We assessed the surface expression of CD19 and CD79A in SEM::EGR3 and SEM::mock using flow cytometry, as the genes encoding these surface receptors were highly ranked and part of the core enrichment of the Reactome BCR signaling gene set (Fig. 2A). Remarkably, although the SEM cell line is per se CD19^+^, EGR3 overexpression resulted in an approximately ten-fold increase of the CD19 median fluorescence intensity (MFI) of the single cell population (*p* < 0.0001) (Fig. 2B, C). Furthermore, we observed a significant relative expansion of the CD19^hi^CD79A^+^ population of SEM::EGR3 (*p* = 0.0006) (Fig. 2D). This expansion of phenotypic B cells (CD19^hi^CD79A^+^) was characterized by a significant increase of the CD79A MFI compared to SEM::mock (*p* = 0.0008) (Fig. 2E).

These results demonstrate that upregulation of *CD19* and *CD79A* by EGR3 led to an increase of the receptors’ surface expressions. EGR3 overexpression induced B-lineage specification indicated by relative expansion of phenotypic B cells, thereby enabling functional BCR signaling as indicated by GSEA.

### EGR3 and downstream intermediate factors transactivate B-lineage specification and commitment genes

To explore the EGR3 regulome in detail, we performed binding and expression target analysis using the open source application BETA plus [24]. The BETA software algorithm integrates transcription factor ChIP-Seq data with differential gene expression data to deduce direct target genes, and is a standard processing pipeline for transcription factor binding studies [24, 32].

BETA enabled integration of MACE-Seq transcriptome data with EGR3 chromatin immunoprecipitation DNA-sequencing (ChIP-Seq) data of SEM::EGR3 and SEM::mock. ChIP-Seq data were already obtained during our former study (GSE205652).

**Fig. 3:**
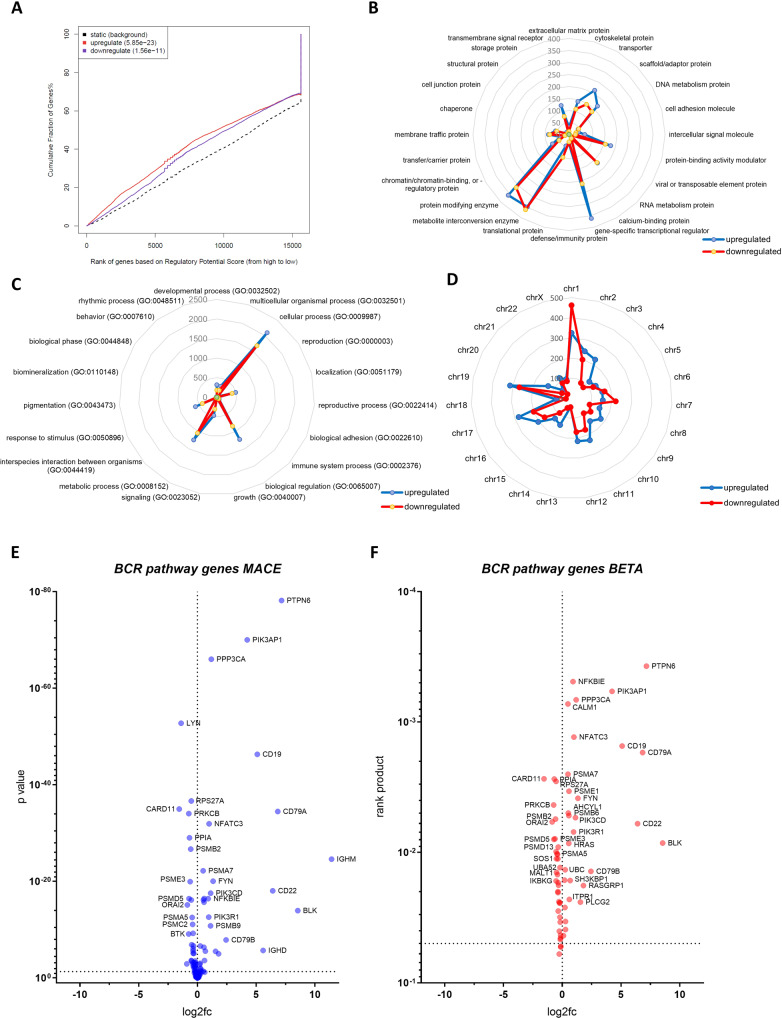
Integration of MACE-Seq and ChIP-Seq data revealed the EGR3 regulome of infant *KMT2A*::*AFF1* ALL. **A** Assignment of gene ranks to the cumulative fraction of genes using BETA identifies EGR3 as a direct activator and repressor of target genes. Analysis of the EGR3 regulome using the PANTHER database with up- and downregulated target genes subclassified considering their corresponding protein class (**B**), biological function/GO term (**C**) and chromosomal location (**D**). Numbers indicate the count of up- (blue) and downregulated (red) genes. **E** Volcano plot visualizing genes of the Reactome BCR geneset according to their differential expression indicated as the log2-fold change (log2fc) and *p* value (MACE-Seq data). **F** Volcano plot visualizing genes of the Reactome BCR geneset according to their differential expression (log2fc) and direct EGR3 regulation (rank product) (MACE-Seq and BETA data).

The BETA algorithm ranked genes according to the regulatory potential score and assigned to the cumulative percentage of genes. Plotting this assignment as a graph visualized that EGR3 owns a direct activating and repressive function, and thus, acts as a direct transactivator and -repressor in *KMT2A*::*AFF1* proB-ALL (Fig. 3A).

In total, 3890 directly upregulated and 3107 downregulated EGR3 target genes were identified, referred to as the EGR3 regulome. We used the PANTHER classification system [25] to functionally characterize the EGR3 regulome. This analysis revealed that EGR3 transactivates and -represses the same classes of genes (Fig. 3B), involved in the same biological functions (Fig. 3C) and located on the same chromosomes (Fig. 3D). Especially the class ‘gene-specific transcriptional regulator’ comprised more directly activated (*n* = 361) than repressed (*n* = 212) genes. Concordant with this, a higher number of transactivated (*n* = 1229) than -repressed (*n* = 840) genes were related to the Gene Ontology (GO) term ‘biological regulation’ (Fig. 3C).

**Fig. 4:**
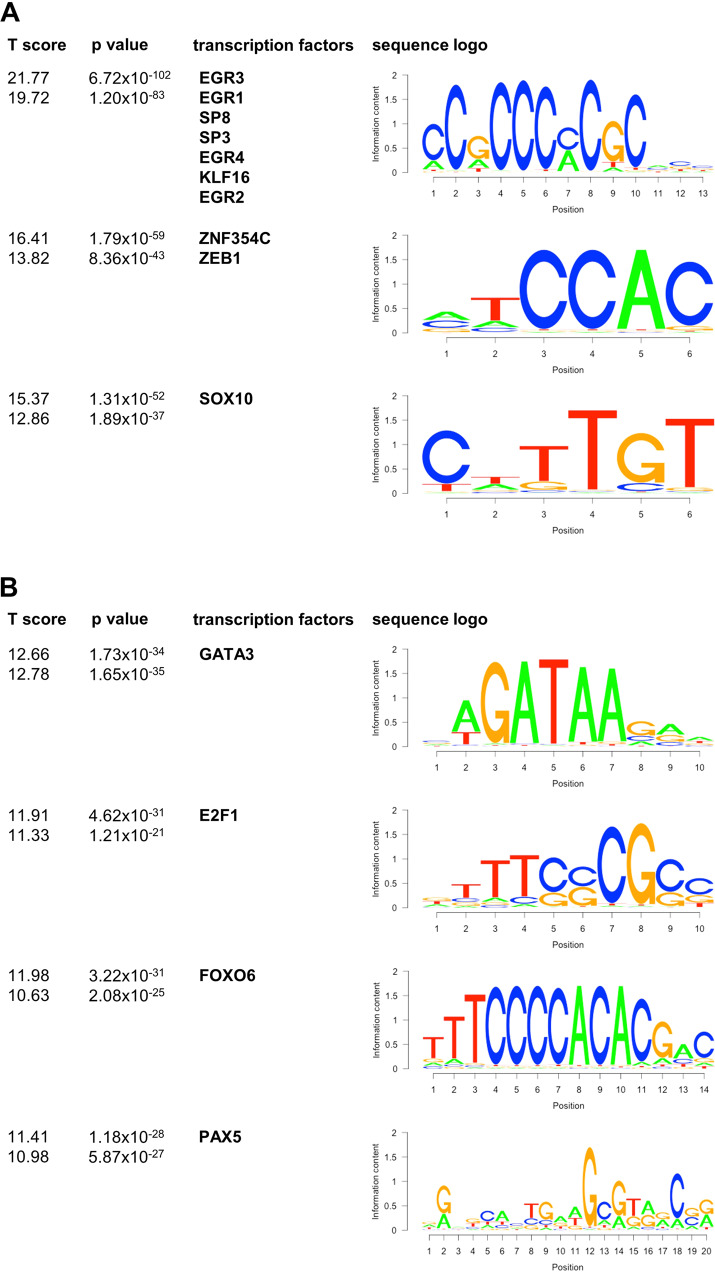
Motif scanning of all differentially expressed genes. **A** Motif scanning result of all direct EGR3 target genes. T score, *p* value, transcription factor and sequence logo were identified using BETA plus. Upper T score and *p* value apply to upregulated genes, lower T score and *p* value apply to downregulated genes. **B** Motif scanning result of differentially expressed but not directly EGR3-regulated genes. T score, *p* value, transcription factor and sequence logo were identified by BETA plus. Upper T score and *p* value apply to upregulated genes, lower T score and *p* value apply to downregulated genes.

We compared the BCR pathway-related genes of the EGR3 transcriptome with those of the regulome (Fig. 3E, F) and identified 64 of 114 genes (56.1%) as direct EGR3 targets, including *CD79A*, *BLK*, *PTPN6*, *CD19*, and *CD22* (Table 6). Thus, the remaining 50 genes including *IGHM* were indirect EGR3 targets and transactivated by unknown intermediate transcription factors. To uncover these transcription factors, a motif analysis of all differentially expressed genes (MACE-Seq data) and all direct EGR3 targets (ChIP-Seq data) was performed using BETA plus. As expected, the highest scoring and significant consensus sequence found in all up- and downregulated target genes matched that one of EGR3 and other transcription factors binding to the same or a highly similar motif including EGR1, EGR2, EGR4, KLF16, SP3, and SP8 (Fig. 4A). Lower scoring binding motifs of up- and downregulated genes were assigned to ZEB1, ZNF354C, and SOX10, indicating potential alternate regulation of EGR3 target genes by these transcription factors. Scanning for enriched motifs in differentially expressed but not direct EGR3-regulated genes identified a set of motif-assigned transcription factors. Of these were *GATA3*, *FOXO6*, and *E2F1* strongly upregulated direct EGR3 targets, and *PAX5* an indirect EGR3 target with strong differential expression (log2fc = 9.47, *p* = 4.95 × 10^−41^) (Fig. 4B). Especially PAX5 has been described as a mediator of B cell identity and B-lineage commitment [33–35]. Furthermore, analysis of the Leukemia MILE study data set using BloodSpot revealed the strongest gene correlations of *PAX5* to be *CD19* and *CD79A*. Concluding this, the binding and expression target analysis followed by motif scanning uncovered GATA3, FOXO6, E2F1, and PAX5 as intermediate factors in the EGR3 transcriptomic network, regulating B-lineage specification and commitment gene expression.

**Table 6:** Genes of the set ‘signaling by the B cell receptor’ (Reactome, R-HSA-983705), their type of regulation by EGR3 and differential expression.

gene	direct EGR3 target?	log2fc MACE-Seq	*p* value MACE-Seq	rp BETA
*IGHM*	no	11.42362217	2.48921E-25	n/a
*BLK*	yes	8.55098816	1.10908E-14	8.46E-03
*PTPN6*	yes	7.164737984	6.50833E-79	0.0003723
*CD79A*	yes	6.842767882	3.2689E-35	0.001712
*CD22*	yes	6.416448966	8.85632E-19	0.006018
*IGHD*	no	5.59443973	1.92888E-06	n/a
*CD19*	yes	5.097472391	4.62783E-47	0.001527
*PIK3AP1*	yes	4.246158952	9.22432E-71	0.0005812
*CD79B*	yes	2.440657291	1.33431E-08	0.01395
*RASGRP1*	yes	1.805660433	9.81104E-06	0.01795
*PLCG2*	yes	1.549262561	3.06887E-06	0.02405
*FYN*	yes	1.335261599	8.88319E-21	0.00384
*PPP3CA*	yes	1.184342322	9.44927E-67	0.0006748
*PIK3CD*	yes	1.124084067	2.75855E-18	0.005402
*PSMB9*	no	1.120899683	1.59578E-11	n/a
*NFATC3*	yes	0.998713529	1.18837E-32	0.001304
*PIK3R1*	yes	0.966311084	2.44716E-13	0.006969
*NFKBIE*	yes	0.914332634	3.75272E-17	0.0004891
*SH3KBP1*	yes	0.677658035	2.64864E-06	0.01642
*PSMA6*	no	0.628450298	0.062177736	n/a
*ITPR1*	yes	0.608990433	0.000233393	0.02294
*PSME1*	yes	0.579774438	3.5101E-17	0.003389
*PSMB6*	yes	0.565638656	2.03686E-16	0.005221
*HRAS*	yes	0.553028624	4.35293E-07	0.008516
*AHCYL1*	yes	0.516916343	6.89158E-17	0.004982
*CALM1*	yes	0.491872592	0.039246507	0.000727
*PSMA7*	yes	0.491383232	6.26317E-23	0.002513
*DAPP1*	no	0.388597531	0.052583747	n/a
*FBXW11*	yes	0.285022076	0.011951906	0.03383
*PPP3CB*	yes	0.27924157	0.031691017	0.03899
*UBC*	yes	0.253791938	2.26985E-07	0.01359
*PSMB8*	no	0.250209285	0.011026312	n/a
*PPP3R1*	yes	0.229316482	0.000922238	0.02644
*PSMC1*	no	0.223758639	0.21214738	n/a
*PSMD8*	yes	0.206878775	0.001486499	0.01632
*NCK1*	no	0.177428109	0.185595712	n/a
*KRAS*	no	0.137742679	0.142867744	n/a
*PSMA8*	no	0.12351416	0.636113579	n/a
*FKBP1A*	yes	0.113168534	0.057549916	0.04344
*PSME2*	no	0.109161404	0.272267991	n/a
*PSMD6*	no	0.099468329	0.227417909	n/a
*BCL10*	no	0.097846109	0.427668399	n/a
*PSMB3*	no	0.093466114	0.130863049	n/a
*PSMA2*	no	0.092612935	0.73577585	n/a
*PSMB11*	no	0.092497922	0.727664144	n/a
*PSMB10*	no	0.091210982	0.717981181	n/a
*PSMB7*	no	0.078594367	0.197210636	n/a
*UBB*	no	0.077804248	0.077068512	n/a
*PSMC6*	no	0.07662523	0.396738012	n/a
*MAP3K7*	no	0.053871719	0.520389098	n/a
*NFATC1*	no	0.017988661	0.842017304	n/a
*BLNK*	no	0.012076394	0.962179165	n/a
*IGLC3*	no	0.005188884	0.98330526	n/a
*PSMC5*	no	0.002298519	0.966883202	n/a
*IGLC2*	no	0	1	n/a
*PSMD2*	no	−0.000438565	0.99385484	n/a
*PSMC3*	no	−0.009374859	0.871548561	n/a
*SEM1*	no	−0.035848586	0.536641158	n/a
*BTRC*	no	−0.043122119	0.743614989	n/a
*NFKBIB*	no	−0.048602913	0.641683594	n/a
*ORAI1*	no	−0.050325263	0.689236497	n/a
*ITPR2*	no	−0.057315013	0.696661836	n/a
*NRAS*	no	−0.067952655	0.403682448	n/a
*PSMC4*	no	−0.090473401	0.188421226	n/a
*CHUK*	no	−0.09213649	0.463552091	n/a
*PSMD14*	no	−0.099389355	0.149921007	n/a
*REL*	no	−0.105755893	0.567292607	n/a
*PSMB5*	yes	−0.117300018	0.071947041	0.0525
*PSMA3*	yes	−0.12765023	0.056413726	0.05304
*PSMA4*	no	−0.12765023	0.056413726	n/a
*STIM1*	no	−0.13504264	0.135062364	n/a
*PSMD4*	yes	−0.136908983	0.043599216	0.04312
*TRPC1*	no	−0.140211415	0.566506534	n/a
*PSMB4*	yes	−0.143316292	0.008444496	0.04541
*SKP1*	yes	−0.155405082	0.007980788	0.04654
*NFATC2*	no	−0.16313576	0.21867056	n/a
*SYK*	no	−0.163962068	0.061590912	n/a
*PSMD3*	yes	−0.170338294	0.002588761	0.03421
*UBA52*	yes	−0.18111628	0.000640896	0.01305
*PSMD10*	no	−0.182449259	0.046626892	n/a
*PSMB1*	yes	−0.191173577	0.000313825	0.03138
*PSMD7*	yes	−0.193336641	0.001402521	0.02428
*RELA*	yes	−0.219182607	0.005520858	0.02449
*PSMF1*	yes	−0.241001349	0.001520813	0.04021
*NFKB1*	no	−0.243914024	0.029935823	n/a
*PSMD9*	yes	−0.25415219	0.084691328	0.06018
*CUL1*	yes	−0.277874069	0.000645415	0.02387
*NFKBIA*	no	−0.284994997	0.008752111	n/a
*GRB2*	yes	−0.310478144	8.1543E-06	0.01782
*PSMD13*	yes	−0.338660552	2.25353E-07	0.009095
*IKBKB*	yes	−0.340568298	0.003982831	0.02814
*ITPR3*	yes	−0.345267237	0.002182622	0.01704
*PSMD1*	yes	−0.355786358	8.26092E-07	0.02033
*PSME4*	no	−0.356370366	7.30394E-06	n/a
*PSMD12*	yes	−0.369060374	2.31513E-06	0.01996
*PSMA1*	yes	−0.370940312	5.60831E-10	0.01119
*VAV1*	yes	−0.396708907	0.000758081	0.01034
*PSMC2*	yes	−0.406420999	8.04357E-12	0.01478
*PSMA5*	yes	−0.44324155	2.8338E-13	0.01024
*MALT1*	yes	−0.479264577	1.28722E-07	0.01421
*SOS1*	yes	−0.486278337	0.000293116	0.01117
*PSMD11*	yes	−0.517998908	7.00864E-17	0.009862
*RPS27A*	yes	−0.518177519	2.15808E-37	0.002853
*IKBKG*	yes	−0.518676703	0.000234099	0.01656
*PSMB2*	yes	−0.565388038	2.01317E-27	0.005551
*PSME3*	yes	−0.610618137	1.09492E-20	0.007852
*PPIA*	yes	−0.673833501	9.15098E-30	0.002734
*PSMD5*	yes	−0.706954282	3.71916E-17	0.007929
*PRKCB*	yes	−0.731810409	8.84403E-35	0.004322
*BTK*	no	−0.742153419	7.76623E-10	n/a
*ORAI2*	yes	−0.847816906	7.16229E-16	0.005828
*RASGRP3*	no	−0.902579249	0.001205017	n/a
*LYN*	no	−1.383822477	1.74706E-53	n/a
*CARD11*	yes	−1.549753074	9.97827E-36	0.002724

*log2fc* log2 fold change, *rp* rank product.

### *IGHM*, *CD79A*, *BLK* and *PTPN6* expressions correlate strongly with each other and partly with *EGR3* among 50 infant *KMT2A*::*AFF1* proB-ALL patients

**Fig. 5:**
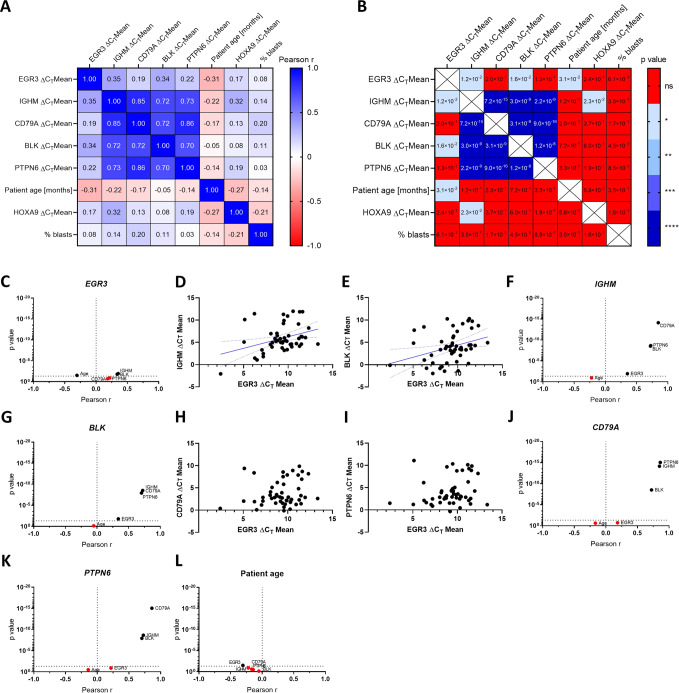
Pearson correlation testing of the diagnosis cohort. Pearson correlation matrix indicating the Pearson *r* value (**A**) and *p* value (**B**) of all tested correlations. Volcano plots showing Pearson correlations of *EGR3* (**C**), *IGHM* (**F**), *BLK* (**G**), *CD79A* (**J**), *PTPN6* (**K**) and patient age (**L**). Correlations with *p* ≥ 0.05 were considered non-significant and marked in red. Patients plotted according to their *IGHM*/*EGR3* (**D**), *BLK*/*EGR3* (**E**), *CD79A*/*EGR3* (**H**) and *PTPN6*/*EGR3* (**I**) ΔC_T_ mean expression values.

We aimed to investigate the regulation of B-lineage specification and commitment gene expression by EGR3 in patient material and therefore assessed the transcription levels of *IGHM, CD79A, BLK*, and *PTPN6* in 50 infant *KMT2A*::*AFF1* proB-ALL patients at diagnosis (Table 1). This patient cohort was already investigated in our recent study [5], from which we obtained the *EGR3* expressions. cDNA of peripheral blood was used for quantitative real-time PCR (qRT-PCR) based gene expression measurement and subsequent Pearson correlation testing of ΔC_T_ values was performed. The resulting Pearson correlation matrix demonstrated a very strong and highly significant correlation between the *IGHM, CD79A, BLK*, and *PTPN6* gene expressions with Pearson r values above 0.70, suggesting their collective belonging to a distinct gene expression program (Fig. 5A, B).

The transcription levels of *EGR3* correlated less strongly with *IGHM* and *BLK* (Pearson *r* = 0.35 and 0.34, respectively) (Fig. 5C–G), and not significantly with *CD79A* and *PTPN6* (Fig. 5C, H–K). Apart from that, higher patient age correlated with higher *EGR3* expression (Pearson *r* = −0.31), whereas this was not the case for *IGHM, CD79A, BLK*, and *PTPN6* (Fig. 5L).

These results confirm the B-lineage phenotype associated with engineered EGR3-overexpression suggesting direct causality between *EGR3* expression and B-lineage specification. Furthermore, other factors are likely to influence the *EGR3* target gene expression as well. The latter conclusion is concordant with the motif scan revealing that EGR1, EGR2, EGR4, KLF16, SP3, SP8, ZEB1, ZNF354C, and SOX10 bind the same genes as EGR3, suggesting these factors as possible co-regulators.

### Low *IGHM*, *CD79A*, *BLK,* and *PTPN6* expressions indicate a patient subgroup with inferior EFS

**Fig. 6:**
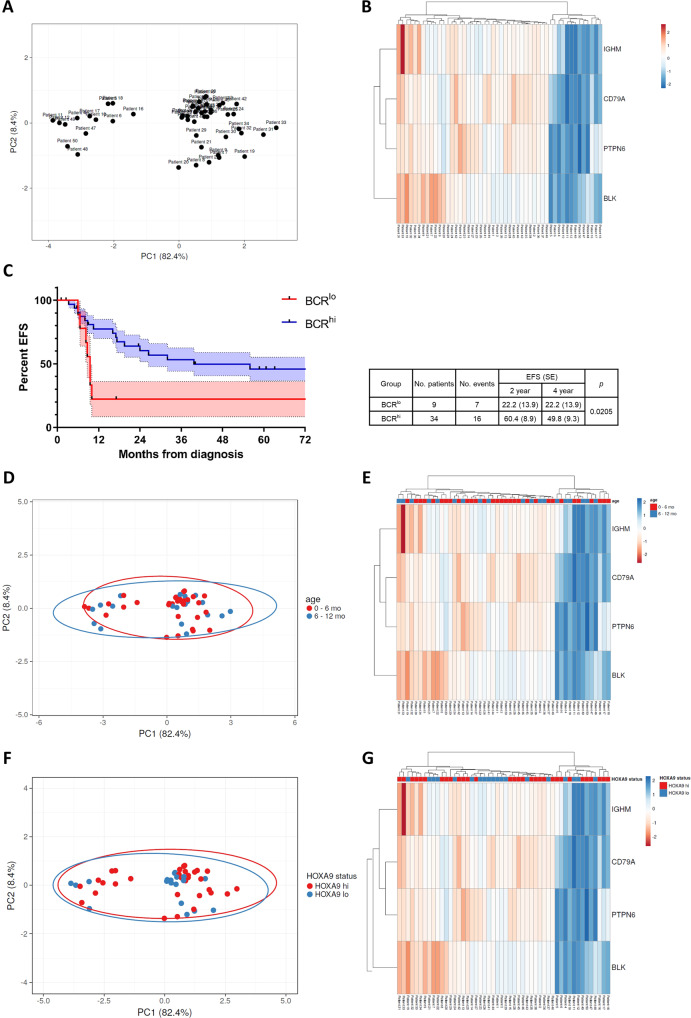
PCA and survival analysis of the diagnosis cohort. **A** PCA of the diagnosis cohort among the *IGHM*, *CD79A*, *BLK*, and *PTPN6* expressions. PC1 represents 82.4% and PC2 8.4% of total variance, respectively. **B** Heatmap of the PCA visualizing bimodal clustering of patients. **C** Kaplan–Meier curves and log-rank test of the BCR^lo^ (red) and BCR^hi^ (blue) patients. S.E.M. of each curve is indicated in bright color. **D** PCA of the diagnosis cohort among the *IGHM*, *CD79A*, *BLK,* and *PTPN6* expressions with patients assigned to the age groups 0–6 months and 6–12 months. **E** Heatmap of the PCA visualizing bimodal clustering of patients is not affected by patient age at diagnosis. **F** PCA of the diagnosis cohort among the *IGHM*, *CD79A*, *BLK,* and *PTPN6* expressions with patients assigned to HOXA status groups *HOXA9* high (hi) and low (lo). **G** Heatmap of the PCA visualizing bimodal clustering of patients is not affected by HOXA status.

The strong correlation between the *IGHM*, *CD79A*, *BLK,* and *PTPN6* gene expressions prompted us to analyze their distribution within the patient cohort. For that purpose, a principal component analysis (PCA) of the expression levels was conducted using the open-source tool ClustVis [22], uncovering a distinct bimodal distribution of patient clusters (Fig. 6A). The corresponding heatmap visualized that patients showed either a high (BCR^hi^, *n* = 37) or low (BCR^lo^, *n* = 13) expression of the analyzed B-lineage-related genes (Fig. 6B). This bimodal distribution points to differences in the B-lineage specification process, with BCR^lo^ patients having a less committed B cell identity, indicated by the low expression of B-lineage-representing genes. Accordingly, the high expression of *IGHM*, *CD79A*, *BLK*, and *PTPN6* in the BCR^hi^ group indicates a more mature proB-cell phenotype. The strict bimodal clustering suggests that the development from BCR^lo^ to BCR^hi^ is rather a stepwise maturation process than a fluent transition, and presumably reflects the developmental stages of early and late proB cells.

We compared the event-free survival (EFS) for all patients with available outcome data (*n* = 43) considering their assignment to the BCR^lo^ (*n* = 9) or BCR^hi^ (*n* = 34) group. Four-year-EFS of BCR^lo^ patients was significantly poorer, reaching only 22.2 ± 13.9% compared to 49.8 ± 9.3% of the BCR^hi^ group (*p* = 0.0205, Fig. 6C). Notably, all events within the BCR^lo^ group occurred within the first 12 months from diagnosis. To test for age and HOXA status as possible confounders, we performed the same PCA analysis with patients assigned to age groups (0–6 months vs. 6–12 months) (Fig. 6D, E) and to *HOXA9* status groups (*HOXA9* high vs. low) (Fig. 6F, G). We observed that both factors did not affect dichotomous separation of patients as young and old as well as *HOXA9* high and low groups overlap almost completely.

### BCR^lo^ vs. BCR^hi^ clustering of patients is sustained at relapse

Considering the elevated level of *EGR3* expression at relapse in infant *KMT2A*-r proB-ALL, we aimed to examine the *IGHM*, *CD79A*, *BLK,* and *PTPN6* gene expressions at the time of relapse. To do so, we performed the same gene expression analysis with the relapse (rel) cohort of our former study. This cohort comprised 18 infant *KMT2A*-r proB-ALL patients (14 *KMT2A*::*AFF1* cases) at the time of relapse, composing an independent cohort, not matched to the diagnosis (dx) cohort (Table 2).

**Fig. 7:**
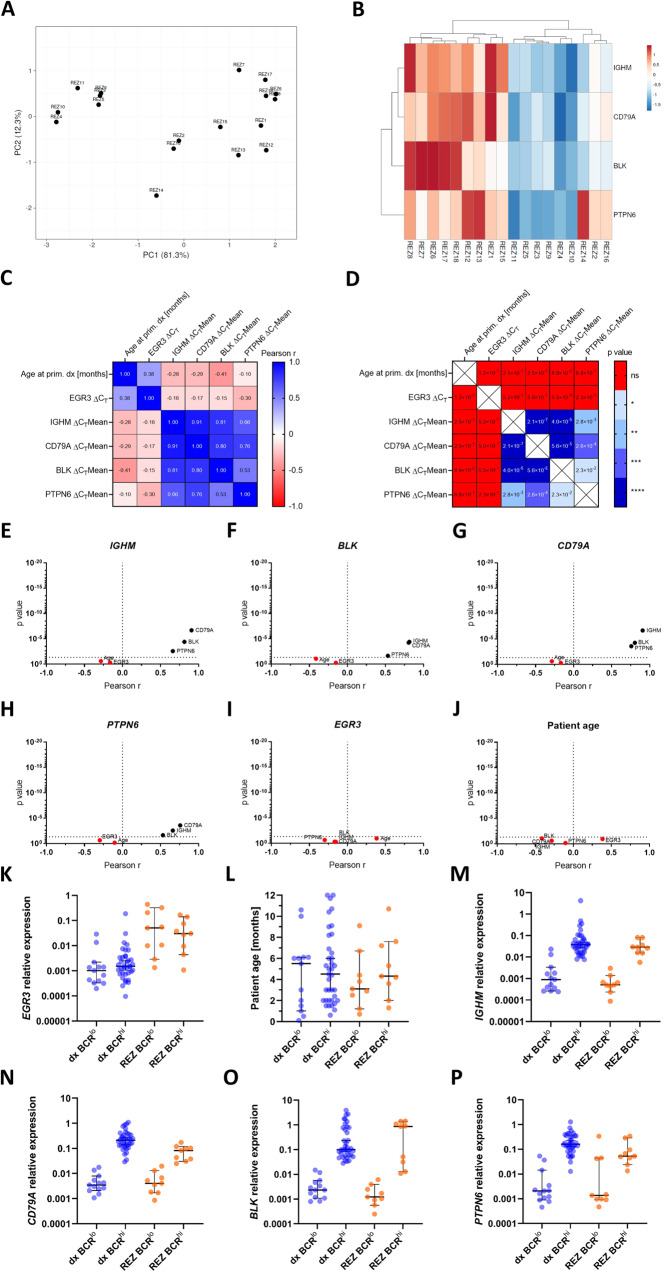
Patient clustering and Pearson correlation testing of the relapse cohort. **A** PCA of the relapse cohort among the *IGHM*, *CD79A*, *BLK,* and *PTPN6* expressions. PC1 represents 81.3% and PC2 12.3% of total variance, respectively. **B** Heatmap of the PCA visualizing clustering of patients. Pearson correlation matrix indicating the Pearson *r* value (**C**) and *p* value (**D**) of all tested correlations. Volcano plots showing Pearson correlations of *IGHM* (**E**), *BLK* (**F**), *CD79A* (**G**), *PTPN6* (**H**) *EGR3* (**I**) and patient age (**J**). Correlations with *p* ≥ 0.05 were considered non-significant and marked in red. Comparison of the BCR^lo^ and BCR^hi^ groups of the diagnosis and relapse cohorts regarding *EGR3* relative expression (**K**), patient age (**L**), *IGHM* (**M**), *CD79A* (**N**), *BLK* (**O**) and *PTPN6* (**P**) relative expressions. Bars indicate the median and 95% confidence interval.

As for the dx cohort, a PCA revealed co-segregation of patients into a BCR^lo^ and a BCR^hi^ group (Fig. 7A). Contrasting the dx cohort, clustering of relapsed patients was less cohesive, with patients REZ2, REZ14, and REZ16 exhibiting low *IGHM*, *CD79A,* and *BLK* expressions, but increased *PTPN6* gene expression levels reaching those of the BCR^hi^ group (Fig. 7B).

Pearson correlation testing of the ΔC_T_ values showed strong correlations between the *IGHM*, *CD79A*, *BLK*, and *PTPN6* gene expressions, almost resembling the Pearson r and corresponding p values of the dx cohort (Fig. 7C–H). Contrasting the dx cohort, the rel cohort did not indicate a significant correlation for any of the aforementioned gene expressions with that of *EGR3* (Fig. 7I). The transcription level of *EGR3* was approximately 100-fold elevated at relapse compared to diagnosis, independent of BCR^lo^ or BCR^hi^ classification (Fig. 7K). Unlike *EGR3*, the expressions of *IGHM*, *CD79A*, *BLK,* and *PTPN6* were not generally elevated at relapse, rather were the expression level differences between the BCR^lo^ and BCR^hi^ groups almost the same comparing the diagnosis and relapse cohorts (Fig. 7M–P). These results indicate that BCR^lo^ vs. BCR^hi^ clustering of patients was sustained at relapse and elevated *EGR3* levels were not concomitant with *IGHM*, *CD79A*, *BLK* and *PTPN6* gene expressions. This suggests a minor role for *EGR3* in B-lineage-specific gene regulation at the time of relapse and demonstrates preservation of both developmental proB cell stages during relapse formation, although to a lesser extent.

## Discussion

This study identified EGR3 as a regulator of B-lineage specification and commitment processes in the context of infant *KMT2A*::*AFF1* acute lymphoblastic leukemia. This characterization is in line with the fact that *EGR3* is generally downregulated in B cell malignancies according to the Leukemia MILE study [27] and upregulated in naïve and mature B cells referring to the Bloodspot DMAP dataset [26] (Supplementary Fig. 1B). Furthermore, the murine homologues Egr3 and Egr2 are required for B cell proliferation upon antigen receptor stimulation [20, 36]. Accordingly, our study and these data imply the limitation of *EGR3* expression to be concomitant with a differentiation block of the B-lineage in hematologic malignancies.

That EGR3 is involved in B-lineage specification and commitment is further strengthened by the identification of PAX5 as an intermediate factor of the EGR3 regulome. PAX5 is a known activator of B cell identity regulating the gene expression of *CD19*, *CD79B,* and *EBF1* [33, 37]. In addition, PAX5 is a regulator of B cell development [38] and B-lineage commitment [35, 39, 40]. In this regard, an important difference between EGR3 and PAX5 is that the latter not only binds DNA response elements but also regulates chromatin accessibility at target promoters [41]. This points to a collaborative effect of EGR3 and PAX5, regulating in part the same genes by different means.

Finally, CD19 is commonly accepted as a hallmark B cell commitment marker [42, 43], and we demonstrate direct transcriptional upregulation of *CD19* by EGR3 resulting in approximately ten-fold increased surface expression of the corresponding protein (Fig. 2). Importantly, CD19-directed therapies including CAR T cells and Blinatumomab were reported to lead to lineage switch of infant *KMT2A*::*AFF1* ALL [44–46]. This process has been shown to be accompanied by a loss of the B-lineage-specific transcription factors EBF1 and PAX5 [47]. As our data suggest a collaborative effect between PAX5 and EGR3, the role of EGR3 during lineage switch of *KMT2A*-r B ALL needs further investigation.

In contrast to primary diagnosis, elevated *EGR3* expression at relapse was not accompanied by increased expression of B lineage-associated direct targets. Consequently, *EGR3* expression at relapse does not indicate a more committed or mature proB cell identity, but could reflect external stress stimuli including response to chemotherapy or inflammatory mediators in the leukemic microenvironment as EGR3 is involved in rapid and transient stress responses and inflammatory signaling [48, 49].

Fetal pre-pro B cells are the earliest B-lineage-committed progenitors giving rise to proB cells [50]. In this context, both progenitor subtypes can be distinguished regarding their expression of B cell-associated genes including *CD79A*, *CD19*, *PAX5*, *MME*, *EBF1*, *DNTT* among many others [50]. As we worked with RNA extracted from peripheral blood, our gene expression analysis considered mainly the blast population. Therefore, it is very likely that clustering of patients into the BCR^lo^ and BCR^hi^ group at diagnosis and relapse represents an early vs. late proB state of the blast population, with BCR^lo^ representing pre-proB cells and BCR^hi^ proB cells. This interpretation is in line with the understanding of B-lineage commitment as a stepwise process, orchestrated by transcription factor-mediated gene regulatory networks [51, 52]. Furthermore, the inferior survival of the BCR^lo^ group could be explained by decreased maturity of blasts within the proB cell state, likely to go along with elevated lineage plasticity, a hallmark of *KMT2A*-r leukemia and implicated in therapy resistance [53, 54]. On the other hand, the undifferentiated phenotype of the BCR^lo^ group could indicate a more aberrant oncogenic signaling program interfering with B-lineage gene expression networks. Nevertheless, the identification of four B-lineage genes whose expression reflects outcome enables early gene expression-based risk-stratification of patients.

That we identified both developmental proB stages at diagnosis and relapse with similar gene expression differences indicates preservation of the blast cell identity from diagnosis through the minimal residual disease phase until relapse formation. Accordingly, it could well be that this cell identity is determined by the cell of origin, which is suggested to belong to the fetal liver-derived hematopoietic progenitor cell compartment [55–57]. However, further studies are needed to uncover mechanisms mediating the developmental arrest of blasts either in the pre-proB or proB stage.

In summary, analysis of the EGR3 regulome of infant *KMT2A*-r iALL identified EGR3 as a regulator of B-lineage commitment. Besides, our study presents four B-lineage genes with prognostic significance, suitable for gene expression-based risk stratification of *KMT2A*-r iALL patients.

## Supplementary information

Supplementary information

Supplementary Figure 1

## Data Availability

All generated datasets are available from the corresponding authors on reasonable request. MACE-Seq data are available at GEO with accession code GSE225710. ChIP-Seq data are available at GEO with accession code GSE205652.
