# Supplementary material for: The EGR3 regulome of infant KMT2A-r acute lymphoblastic leukemia identifies differential expression of B-lineage genes predictive for outcome
Source: Leukemia. 2023 Apr 26;37(6):1216–33. doi: 10.1038/s41375-023-01895-z (PMC10132433; doi:10.1038/s41375-023-01895-z)
Supplement: Supplementary file 1 — Supplementary information [file 41375_2023_1895_MOESM1_ESM.docx]

**Supplementary information to 22-LEU-1729R**

Külp et al., The EGR3 regulome of infant KMT2A-r acute lymphoblastic leukemia identifies differential expression of B-lineage genes predictive for outcome

**Supplementary Figure 1: EGR3 expression under leukemic and healthy conditions**

**A** EGR3 log2 expression values among different leukemia subtypes. Data of the Leukemia MILE study (n=2096 patients) (27), analyzed and visualized using the BloodSpot database (26).

**B** Relative EGR3 expression in the hematopoietic system. Data of the DMAP dataset (28), analyzed and visualized using the BloodSpot database (26).
