# Supplementary figures and images for: The EGR3 regulome of infant KMT2A-r acute lymphoblastic leukemia identifies differential expression of B-lineage genes predictive for outcome
Source: Leukemia. 2023 Apr 26;37(6):1216–33. doi: 10.1038/s41375-023-01895-z (PMC10132433; doi:10.1038/s41375-023-01895-z)

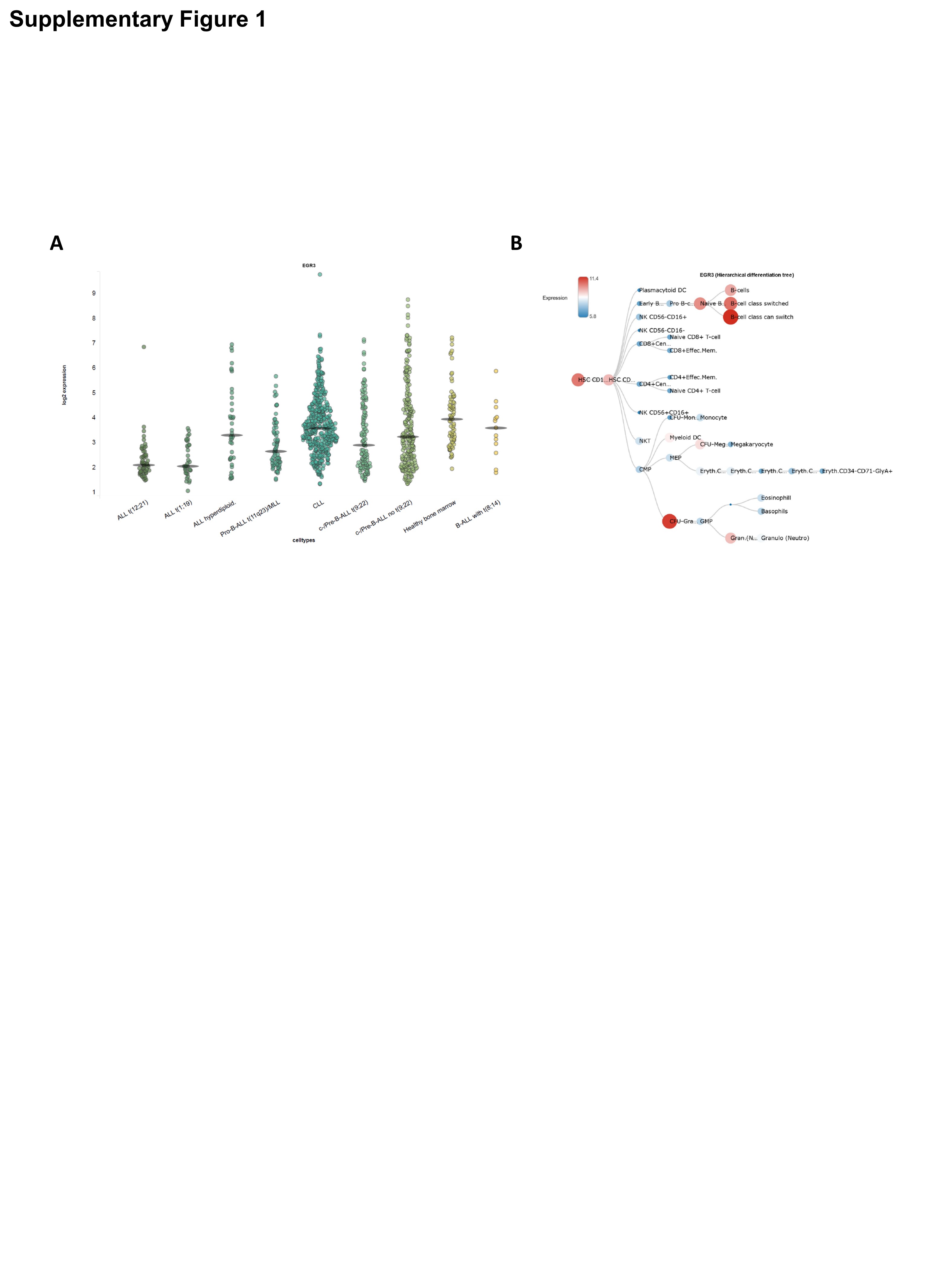

Supplement: Supplementary file 2 — Supplementary Figure 1 [file 41375_2023_1895_MOESM2_ESM.jpg]
